# Trimodal prehabilitation for hematopoietic stem cell transplantation: A best evidence summary

**DOI:** 10.1016/j.apjon.2026.100993

**Published:** 2026-06-12

**Authors:** Danni Wu, Limei Xue, Lingzhi Xu, Jie Pan, Tao Chen, Yan Wang, Sufang Zhao

**Affiliations:** Department of Hematology, The First Affiliated Hospital of Soochow University, Suzhou, China

**Keywords:** Hematopoietic stem cell transplantation, Prehabilitation, Hematological malignancies, Evidence-based summary

## Abstract

**Objective:**

To systematically evaluate and synthesize the best practice evidence of trimodal prehabilitation comprising exercise, nutritional optimization, and psychological support in patients undergoing hematopoietic stem cell transplantation, so as to enhance patients’ physiological reserve and stress resistance, and provide a scientific basis and practical reference for formulating individualized prehabilitation programs in clinical practice.

**Methods:**

A comprehensive computerized literature search was conducted across multiple databases and evidence sources, including UpToDate, BMJ Best Practice, Guidelines International Network, National Institute for Health and Care Excellence, Scottish Intercollegiate Guidelines Network, National Guideline Clearinghouse, Registered Nurses Association of Ontario, Joanna Briggs Institute, World Health Organization, Cochrane Library, Web of Science, PubMed, CINAHL, China National Knowledge Infrastructure, Wanfang Data, and SinoMed. The search timeframe encompassed literature from September 2015 to September 2025.

**Results:**

A total of 11 publications were included, comprising 1 clinical guideline, 2 expert consensus documents, 1 evidence summary, 3 systematic reviews, and 4 randomized controlled trials. The evidence was synthesized across five domains: multidisciplinary team building, exercise prescription, nutritional optimization, psychological support, and precautionary considerations, resulting in the formulation of 34 distinct evidentiary statements.

**Conclusions:**

This study synthesizes the best available evidence on trimodal prehabilitation for patients undergoing hematopoietic stem cell transplantation, providing an evidence-based foundation for standardizing pre-transplant rehabilitation management. It is recommended that healthcare professionals, when implementing these recommendations, comprehensively consider the specific clinical context to selectively apply the most appropriate evidence, with the aim of improving patient transplant outcomes.

**Systematic review registration:**

This study was registered at the Fudan University Center for Evidence-Based Nursing (Registration No. ES20259181).

## Introduction

Hematopoietic stem cell transplantation (HSCT) is a critical therapeutic strategy for reconstituting a recipient’s hematopoietic and immune system. This is typically achieved by administering high-dose chemotherapy or radiotherapy to eradicate abnormal hematopoietic cells, followed by the infusion of hematopoietic stem cells.^1^ Currently, this procedure has become a cornerstone therapeutic approach for various hematologic malignancies, including lymphoma, acute leukemia, and multiple myeloma, as well as for certain genetic disorders, solid tumors, and autoimmune diseases. The annual global number of autologous or allogeneic transplants performed now exceeds 60,000.^2^^,^^3^

Despite its significant improvement in disease control and overall survival rates, the therapeutic benefits of HSCT remain influenced by multiple factors, including recipient age, baseline disease burden, comorbidities, and human leukocyte antigen (HLA) compatibility between donor and recipient. Furthermore, the treatment process is frequently associated with multi-system complications.^4^^,^^5^ Among the various factors influencing prognosis, the patient’s nutritional status and functional reserve have increasingly been demonstrated to hold significant predictive value.^3^ Studies indicate that patients with low or excessive body weight prior to transplantation exhibit lower long-term survival rates post-transplant. Similarly, physical performance status, as a functional indicator, reflects a patient’s tolerance to intensive treatment. Furthermore, treatment-related physical side effects may exacerbate psychological issues such as anxiety and depression, which in turn can negatively impact quality of life and the recovery trajectory.^3^^,^^6^^,^^7^

However, data indicate that the physical reserve status of HSCT patients prior to treatment is suboptimal. Research by Chmielewski M et al. found that pre-transplant patients’ physical activity levels are generally below the recommended standards set by the World Health Organization (WHO) and the American College of Sports Medicine (ACSM).^8^ The prevalence of malnutrition prior to transplantation ranges from 10% to 50%.^9^; Furthermore, 41% of patients experience severe fatigue within five years following the transplantation procedure.^3^ Notably, even among patients with initially adequate physical fitness upon hospital admission, studies have observed a substantial decline in walking distance from admission to discharge. This finding suggests that the entire HSCT process imposes significant functional depletion on the body.^6^

Therefore, optimizing pre-transplant management is of significant importance for improving postoperative survival in transplant patients. To address this gap, the concept of trimodal prehabilitation has been increasingly applied to the pre-transplant management of patients undergoing HSCT. This approach, which integrates exercise training, nutritional optimization, and psychological support into a coordinated program initiated prior to treatment, was originally proposed by Li et al.^10^ in the setting of oncologic surgery. By enhancing the patient’s physical and psychological status preoperatively, it aims to improve surgical tolerance and create a foundation for accelerated postoperative recovery. Prehabilitation has been widely adopted in fields such as solid organ transplantation and oncologic surgery and is considered effective in reducing postoperative complications, promoting functional recovery, and shortening hospital length of stay.^11^^,^^12^ Given the profound multisystem stress responses, prolonged immunosuppression, and the impact of enforced isolation associated with the HSCT process, patients undergoing HSCT may experience synergistic declines in physical, nutritional, and psychological domains.^13^ The recovery period for these patients can last from weeks to months, during which functional deficits in one area can exacerbate impairments in others; therefore, the concurrent optimization of these three intervention components prior to treatment is an essential measure to help patients build resilience and withstand treatment-related toxicities. Given their continuous involvement throughout the transplant care continuum, nurses are uniquely positioned to lead and coordinate comprehensive pre-transplant interventions that integrate physical, nutritional, and psychosocial support.^14^ However, the management of HSCT patients by nursing staff is frequently based on clinical experience, without unified standards regarding the timing of dietary education, the intensity and frequency of exercise, and psychological guidance. There is a lack of authoritative and detailed guidelines for trimodal prehabilitation management.^15^ The overwhelming volume of information may lead to uncertainty and stress among nursing staff. Relying solely on clinical experience without scientifically grounded practical guidance is not an optimal approach for managing patient preparation prior to HSCT.^15^^,^^16^

Several studies have investigated prehabilitation in HSCT patients. For example, research by Liang et al.^3^ demonstrated that initiating exercise training before HSCT is more effective in improving patients’ functional levels compared to starting after transplantation. Dennett et al.^17^ found that impaired nutritional status prior to and during transplantation can be potentially modulated and improved through early nutritional support. Furthermore, studies by Chen et al.^18^ indicated that common psychosocial interventions, including psychosocial counseling, psychoeducation, cognitive-behavioral therapy (CBT), and mindfulness-based stress reduction, are beneficial for fatigue management in HSCT patients. They recommend combining these interventions with exercise to reduce fatigue levels. These findings collectively suggest that a comprehensive pre-transplant trimodal prehabilitation program, integrating structured physical activity, psychological support, and nutritional management, should constitute an essential component of nursing care.

Unfortunately, evidence supporting the implementation of trimodal prehabilitation in HSCT patients remains limited. Existing HSCT-related guidelines primarily focus on transplantation procedures, pharmacotherapy, and complication management, with insufficient attention paid to prehabilitation.^19^ Although guidelines for nutritional management in HSCT patients include some recommendations for pre-transplant nutritional interventions, they rarely integrate exercise or psychological components. Second, exercise interventions are currently only supported by preliminary studies, with no standardized recommendation schemes available. Finally, evidence related to psychosocial care during the peri-transplant and post-transplant periods is lacking, and little evidence extends to the prehabilitation phase.^17^^,^^20^ Importantly, no guidelines or systematic reviews have been retrieved that integrate exercise, nutrition, and psychological support into a unified pre-transplant trimodal framework specifically for HSCT recipients, which has hindered its clinical promotion and practical application.

Despite the promising potential of trimodal prehabilitation, a critical evidence-to-practice gap hinders its implementation in HSCT nursing care. To bridge this gap, the present study systematically identifies, appraises, and synthesizes the best available evidence to generate a set of structured, nursing-oriented recommendations for trimodal prehabilitation in HSCT, with the aim of guiding clinical decision-making, supporting protocol development, and facilitating consistent, evidence-based prehabilitation delivery by nursing teams.

## Methods

### Question identification

Based on the PIPOST framework, the clinical question was translated into an evidence-based inquiry: (1) P (Population): patients aged ≥ 18 years undergoing HSCT; (2) I (Intervention): measures related to trimodal prehabilitation involving exercise training, nutritional management, and psychological support prior to transplantation, including assessment and nursing care; (3) P (Professionals): healthcare professionals; (4) O (Outcomes): transplantation-related complications, relevant hematological parameters, exercise capacity, muscle strength, nutritional status, health-related quality of life, cancer-related fatigue levels, adherence, and satisfaction; (5) S (Setting): healthcare environments; (6) T (Type of Evidence): clinical decisions, guidelines, expert consensus documents, practice recommendations, meta-analyses, systematic reviews, evidence summaries, best practice statements, and randomized controlled trials (RCTs).

### Retrieval strategy

Using the “6S” evidence hierarchy model, a top-down approach was adopted to retrieve relevant literature.^21^ We prioritized the retrieval of the highest-level evidence to ensure the first acquisition of authoritative and integrated high-quality evidence; when high-level evidence was insufficient, we supplemented the retrieval with original studies, so as to improve the efficiency and quality of evidence retrieval and avoid the omission of important evidence. We searched professional association websites and guideline repositories using terms such as “hematopoietic stem cell transplantation” and “prehabilitation”. For database searches, we employed a combination of subject headings and free-text terms. The following databases were searched: UpToDate, BMJ Best Practice, Guidelines International Network (GIN), National Institute for Health and Care Excellence (NICE), Scottish Intercollegiate Guidelines Network (SIGN), National Guideline Clearinghouse (NGC), Registered Nurses Association of Ontario (RNAO), Joanna Briggs Institute (JBI), World Health Organization (WHO), Cochrane Library, Web of Science, PubMed, CINAHL, China National Knowledge Infrastructure (CNKI), Wanfang Data, and SinoMed. The detailed search strategies are available in Supplementary File 1.

### Literature inclusion and exclusion criteria

#### Literature inclusion criteria

(1) Participants: patients aged ≥ 18 years undergoing HSCT. (2) Content: studies addressing rehabilitation management before HSCT, including both single-modality intervention studies and multi-modality combined intervention studies. (3) Study types: guidelines, expert consensus statements, expert opinions, practice recommendations, meta-syntheses, meta-analyses, systematic reviews, best practice statements, clinical decisions, and RCTs. (4) To ensure evidence timeliness, only literature published between September 2015 and September 2025 was included.

#### Literature exclusion criteria

(1) Literature types such as guideline interpretations, guideline impact evaluations, research protocols, or proposals. (2) Updated versions of guidelines superseded by newer editions. (3) Duplicate publications or literature with unavailable full text. (4) Literature of low methodological quality. (5) RCTs already included in existing guidelines, evidence summaries, or systematic reviews.

### Literature quality evaluation standard

To ensure scientific rigor, standardization, and specificity of literature quality assessment, this study strictly selected appropriate professional assessment tools according to evidence type:^22^ (1) Clinical guidelines were evaluated using the Appraisal of Guidelines for Research and Evaluation Instrument II (AGREE II).^23^ AGREE II comprises 6 domains with a total of 23 key items. Agreement with each item was rated on a seven-point scale (1 = strongly disagree to 7 = strongly agree). Domain scores were standardized as percentages of the maximum possible score. Guidelines with ≥ 6 domains scoring ≥ 60% were assigned a recommendation level of A; those with 3–5 domains ≥ 60% were rated B; all others were rated C. (2) Expert consensus documents were assessed using the Joanna Briggs Institute (JBI) Critical Appraisal Checklist for Text and Opinion Papers. Each item was judged as “yes”, “no”, “unclear”, or “not applicable”.^24^ (3) Systematic reviews were appraised with the JBI Critical Appraisal Checklist for Systematic Reviews and Research Syntheses (2016 edition), using the categories “yes”, “no”, “unclear”, or “not applicable”.^25^ (4) Evidence summaries and practice recommendations were evaluated with the Critical Appraisal of Summarized Evidence (CASE) tool, which includes 10 items rated as “yes”, “no”, or “partially”.^26^ (5) RCTs were assessed using the JBI Critical Appraisal Checklist for RCTs.^27^ Two researchers independently conducted the quality assessment according to the inclusion and exclusion criteria. Any discrepancies were resolved through discussion among the evidence-based working group. All of them had completed formal training in evidence-based nursing methodology. Following the quality assessment, the methodological strengths and limitations of each included study were analyzed based on the quality evaluation results.

### Evidence extraction and integration

The evidence-based team synthesized the evidence according to the following principles: (1) When the content was consistent across sources, the more clearly understandable statement was selected. (2) When multiple sources presented complementary information, the content was logically and linguistically integrated into a single evidence statement. (3) In cases of conflicting conclusions from different sources, priority was given to evidence-based sources, higher-quality evidence, and the most recently published authoritative literature. The JBI Evidence Pre-JBI Levels of Evidence and Grades of Recommendation system (2014 edition) was used to grade the evidence and determine recommendation levels. The grading criteria are as follows: Level 1-RCTs or meta-analyses of RCTs; Level 2-quasi-experimental studies; Level 3-observational analytical studies; Level 4-observational descriptive studies; Level 5-expert opinion and basic research.^28^ The evidence level is determined by the highest-quality literature that directly supports each recommendation statement. Subsequently, an expert review meeting was convened. The panel comprised two hematologists (both associate chief physicians with ≥ 10 years of clinical experience), one head nurse of the hematology department (associate chief nursing officer with ≥ 20 years of experience), and four nursing team leaders from the hematology department (one associate chief nursing officer and three supervisory nurses, all with ≥ 10 years of experience). Initially, panel members independently reviewed the evidence and corresponding source literature. They then scored each evidence item based on the FAME principles (Feasibility, Appropriateness, Meaningfulness, and Effectiveness), evaluating factors such as the difficulty of clinical implementation, alignment with the clinical context of HSCT in China, potential value for patient prognosis, and the strength of the evidence base. Following the collection and aggregation of these scores, items with divergent opinions were discussed individually to reach a consensus through negotiation. Ultimately, the panel jointly determined the recommendation strength for each evidence statement, categorizing them into two levels: Strong Recommendation (Grade A) and Weak Recommendation (Grade B).

## Results

### Literature search results and general information

**Fig. 1:**
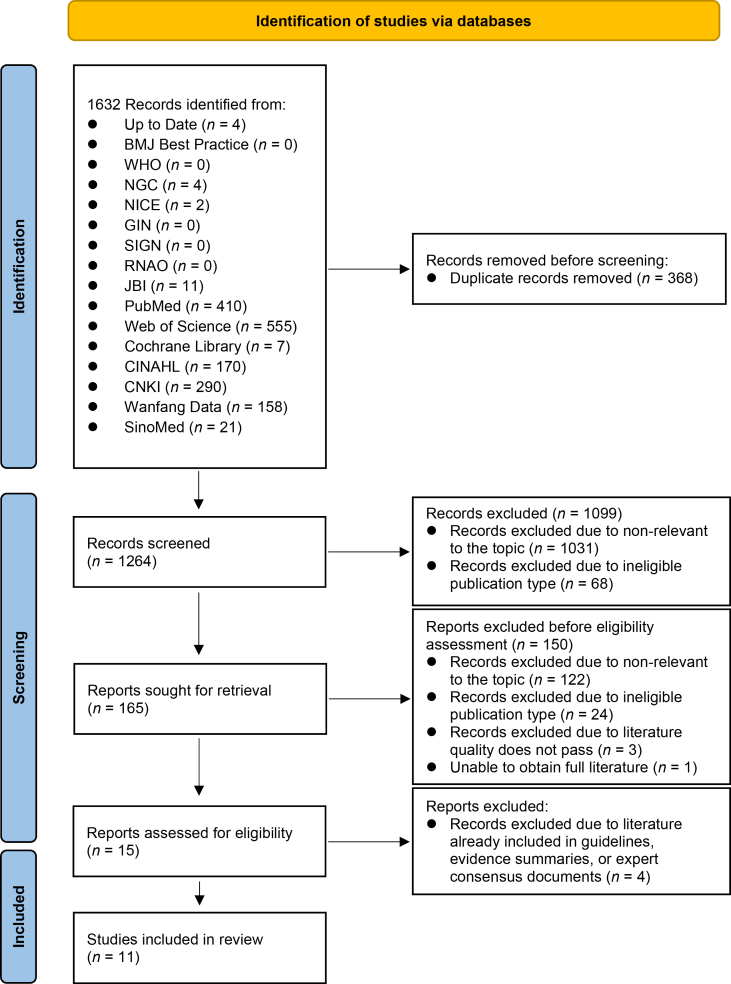
Screening flow chart for literature.

A total of 1632 records were identified through the search. After deduplication and screening of titles, abstracts, and full texts, 11 publications were ultimately included. Fig. 1 illustrates the literature selection process following the PRISMA flow diagram. The final set comprised 1 clinical guideline, two expert consensus documents, 1 evidence summary, 3 systematic reviews, and 4 RCTs. The basic characteristics of the included literature are presented in Table 1.^2^, ^5^, ^17^, ^18^, ^20^, ^29^, ^30^, ^31^, ^32^, ^33^, ^34^

**Table 1:** Evidence source and content (*N* = 11).

Author	Literature source	Literature type	Publication/Update date	Topic
Pinelli et al.^29^	SFC	Guideline	2025	Rehabilitation across the pre-, peri-, and post-transplantation phases in HSCT patients
Barban et al.^2^	SBTMO	Expert consensus	2020	Nutrition management for HSCT patients
Chinese Society of nutritional oncology^30^	CNKI	Expert consensus	2024	Nutrition therapy for bone marrow transplant patients
Chen et al.^18^	CNKI	Evidence summary	2023	Fatigue management in HSCT patients
Morales-Rodriguez et al.^5^	PubMed	Systematic review	2022	The impact of exercise on physical function and safety in HSCT patients
Baliousis et al.^31^	PubMed	Systematic review	2016	Psychological intervention for HSCT patients
Baumgartner et al.^32^	PubMed	Systematic review	2017	Nutritional support for HSCT patients
Potiaumpai et al.^20^	PubMed	RCT	2024	Exercise intervention prior to HSCT
Dennett et al.^17^	PubMed	RCT	2025	Multidisciplinary prehabilitation for HSCT patients
An et al.^33^	PubMed	RCT	2023	HSCT patient exercise program
Wood et al.^34^	PubMed	RCT	2020	Home-based exercise prescription prior to HSCT

HSCT, hematopoietic stem cell transplantation; RCT, randomized controlled trial; SBTMO, The Brazilian Society of Bone Marrow Transplantation; SFC, Société Française du Cancer.

### Literature quality-evaluation results

#### Guidelines quality evaluation

This study included one guideline from the French Cancer Society, which was assigned a B-grade recommendation and subsequently incorporated. This guideline had a clear scope and purpose, a transparent development process, and an independent writing team; however, its the rigor of development was limited, which may compromise the operability of some recommendations in clinical practice. Details are presented in Table 2.

**Table 2:** Guidelines quality evaluation results (*N* = 1).

Guideline	Normalized percentage of scores (%)						≥ 60% of domains	≥ 30% of domains	Recommendation level
	Scope and Purpose	Involved personnel	Preciseness of guideline development	Clarity of presentation	Applicability	Independence of writing			
Pinelli et al.^29^	94.4	83.3	52.1	77.8	87.5	83.3	5	6	B

Recommendation level: Grade A, ≥ 6 domains with scores ≥ 60%; Grade B, 3–5 domains with scores ≥ 60%; Grade C, others.

#### Expert consensus quality evaluation

One expert consensus document from the Brazilian Society of Bone Marrow Transplantation and one from the Chinese Society of Nutritional Oncology were included. Both documents provided substantial content and were incorporated. The included expert consensus demonstrated consistent methodological strengths: clear sources of opinions and content focused on the core interests of patients. The details are presented in Table 3.

**Table 3:** Quality evaluation results of the included expert consensus (*N* = 2).

Include literature	①	②	③	④	⑤	⑥
Barban et al.^2^	Yes	Yes	Yes	Yes	Yes	Unclear
Chinese Society of nutritional oncology^30^	Yes	Yes	Yes	Yes	Yes	Yes

①Is the source of the opinion clearly identified? ②Does the source of opinion have standing in the field of expertise? ③Are the interests of the relevant population the central focus of the opinion? ④Does the opinion demonstrate a logically defended argument to support the conclusions drawn? ⑤Is there reference to the extant literature?⑥Is any incongruence with the literature/sources logically defended?

#### Systematic reviews quality evaluation

A total of three systematic reviews were included in the study. These reviews were characterized by rigorous and well-structured designs and were therefore incorporated. Overall acceptable methodological quality, although one review did not assess publication bias. The relevant details are presented in Table 4.

**Table 4:** Quality evaluation results of the included systematic reviews (*N* = 3).

Include literature	①	②	③	④	⑤	⑥	⑦	⑧	⑨	⑩	⑪
Morales-Rodriguez et al.^5^	Yes	Yes	Yes	Yes	Yes	Yes	Yes	Yes	NO	Yes	Yes
Baliousis et al.^31^	Yes	Yes	Yes	Yes	Yes	Yes	Yes	Yes	Yes	Yes	Yes
Baumgartner et al.^32^	Yes	Yes	Yes	Yes	Yes	Yes	Yes	Yes	Yes	Yes	Yes

① Is the evidence-based question raised clear and explicit? ② Is the inclusion criteria for literature appropriate for this evidence-based question? ③ Is the retrieval strategy appropriate? ④ Is the search database or resources sufficient? ⑤ Is the literature quality evaluation standard used appropriate? ⑥ Are there two or more evaluators independently completing quality evaluations? ⑦ Are certain measures taken to reduce errors when extracting data? ⑧ Is the method of merging research appropriate? ⑨ Has the possibility of publication bias been evaluated? ⑩ Are the policy or practice recommendations based on the results of a systematic evaluation? ⑪ Is the proposed further research direction appropriate?

#### Evidence summary quality evaluation

One evidence summary published by the Chinese Nursing Association was included. This document provided comprehensive content and was incorporated into the analysis. This evidence summary provided relevant support. The details are presented in Table 5.

**Table 5:** Quality evaluation results of the included evidence summary (*N* = 1).

Include literature	①	②	③	④	⑤	⑥	⑦	⑧	⑨	⑩
Chen et al.^18^	Yes	Yes	Yes	Yes	Yes	Yes	Yes	Yes	Yes	Yes

① Specificity of the application scope and target audience; ② Transparency of the author’s identity; ③ Transparency of the review process; ④ Clarity of evidence grading; ⑤ Clarity of recommendation statements; ⑥ Appropriateness of citation for recommendation statements; ⑦ Timeliness of recommendation statements; ⑧ Applicability for the evaluation of the study population; ⑨ Comprehensive nature of the literature search; ⑩ Disclosure of potential conflicts of interest.

#### Randomized controlled trial quality evaluation

This study incorporated four RCTs. The included RCTs exhibited favorable methodological strengths. The main limitations were small sample sizes and inadequate handling of missing data in some studies, which may increase the risk of implementation bias in the results. The results of their quality assessment are presented in Table 6.

**Table 6:** Quality evaluation results of the included randomized controlled trials (*N* = 4).

Include literature	①	②	③	④	⑤	⑥	⑦	⑧	⑨	⑩
Potiaumpai et al.^20^	Yes	NO	Yes	Unclear	NO	Yes	Yes	Yes	Yes	Yes
Dennett et al.^17^	Yes	Yes	Yes	Unclear	Yes	Yes	Yes	Yes	Yes	Yes
An et al.^33^	Yes	Yes	Yes	NO	NO	Yes	Yes	Yes	Yes	Yes
Wood et al.^34^	Yes	Yes	Yes	Yes	Yes	Unclear	Unclear	Yes	Yes	Yes

1 Implementation of random allocation methods; ② application of blinding to study participants; ③ execution of allocation concealment; ④ handling of participants with missing data; ⑤ blinding of outcome assessors; ⑥ baseline comparability; ⑦ consistency of intervention implementation; ⑧ consistency of measurement methods; ⑨ reliability of the measurement personnel; ⑩ appropriateness of data analysis methods.

### Evidence summary and description

The evidence was synthesized across five domains, multidisciplinary team building, exercise prescription, nutritional optimization, psychological intervention, and precautionary considerations, resulting in the formulation of 34 evidentiary statements as presented in Table 7. The evidence recommendations are dominated by Grade A strong recommendations and supplemented by Grade B recommendations.

**Table 7:** Trimodal prehabilitation in patients undergoing hematopoietic stem cell transplantation.

Evidence topic	Evidence Content	Evidence Level	Recommendation
Multidisciplinary team building	1. Establish a specialized pre-transplant rehabilitation management team comprising physicians, physical therapists, dietitians, psychologists, and specialized nurses. The team will convene meetings at regular intervals to dynamically adjust the exercise, nutritional, and psychological intervention plans, thereby ensuring the synergistic effect of the interventions.^17^^,^^29^	Level 1	A
	2. The implementation of guideline-based assessment and intervention design by trained clinical staff can enhance the safety of pre-transplant rehabilitation.^17^	Level 1	A
	3. Pre-transplant rehabilitation should be initiated as early as possible and implemented no later than the formal confirmation of the transplantation indication.^18^^,^^29^	Level 1	A
Exercise prescription	4. A physical therapist should conduct the assessment and provide standardized written instructions that include exercise guidelines.^17^	Level 1	A
	5. The assessment should encompass the patient’s age, prior exercise level, disease treatment regimen, medication history, family history, routine clinical parameters, exercise capacity, mobility impairments, and contraindications.^17^^,^^20^^,^^33^	Level 1	A
	6. A registered exercise physiologist with specialized oncology training should tailor the exercise program for each participant based on their fitness status, clinical history, and lifestyle.^5^^,^^33^	Level 1	B
	7. Contraindications to exercise for HSCT patients include acute bleeding, platelet count < 10 × 10^9^/L, hemoglobin concentration < 80 g/L, severe pain, body temperature ≥ 38°C, active severe infection, altered mental status, dizziness, nausea, and vomiting. When developing an exercise program, the safety and feasibility of physical activity should be evaluated in light of the patient’s condition and the clinical context.^18^	Level 1	B
	8. The principle of gradual progression should be followed, with exercise duration and intensity increased stepwise according to the patient’s tolerance.^5^^,^^29^^,^^33^	Level 1	A
	9. Exercise modalities should include warm-up, aerobic exercise, resistance training, stretching, and cool–down activities.^17^^,^^20^ Resistance training may include bodyweight exercises for the upper and lower limbs, free-weight training, and elastic band exercises. Aerobic exercise can encompass walking or the use of a portable stepper.^17^^,^^18^	Level 1	B
	10. Exercise intensity: Exercise intensity can be assessed using various methods during the activity. For example, intensity may be evaluated based on the patient’s heart rate, with a target range of 50%–80% of the predicted maximum heart rate. The predicted maximum heart rate (beats per minute) is calculated as 220 minus the patient’s age. Alternatively, the modified Borg scale is recommended for monitoring perceived exertion.^5^^,^^17^^,^^18^^,^^29^^,^^33^	Level 1	B
	11. The feasibility of implementing home-based high-intensity aerobic interval training is low.^34^	Level 1	B
	12. The monitoring procedures must be designed to be as patient-friendly as possible to encourage broad participation.^34^	Level 1	B
	13.Patients with unstable bone lesions require close supervision by exercise professionals and are better suited to receive treatment within a healthcare facility.^17^	Level 1	B
Nutritional Optimization	14. It is recommended to initiate dietary management before transplantation and to maintain it throughout all phases and modalities of HSCT. This ensures the appropriate provision of nutrients and calories, thereby improving the patient’s capacity to tolerate treatment.^2^^,^^32^	Level 1	A
	15. Nutritional management must be clearly defined and is recommended to be established in collaboration with the patient and their caregivers, as they are directly involved in dietary care.^2^	Level 5	A
	16. A dietitian should provide written materials and personalized medical nutrition therapy to the patient via telephone or video call every two weeks prior to transplantation.^17^	Level 1	B
	17. It is recommended to conduct a pre-transplant nutritional assessment within 30 days before admission for HSCT. The aim is to restore or maintain the patient’s nutritional status prior to transplantation and to correct any nutritional deficiencies, thereby minimizing the adverse impact of HSCT on nutritional health.^2^^,^^30^	Level 5	B
	18. The frequency of nutritional assessment should be determined based on the patient’s nutritional risk status. For outpatients already identified with nutritional risk, the assessment interval should not exceed 15 days. For patients without current nutritional risk, the assessment interval may be extended to 30 days.^2^	Level 5	A
	19. Inpatient screening must be completed within 48 hours of admission, followed by a reassessment 7 days later. Subsequent reassessments should then be conducted weekly until the patient is discharged home.^2^	Level 5	A
	20. The most suitable tool for assessing nutritional risk in inpatients is the nutritional Risk screening 2002 (NRS-2002).^2^	Level 5	A
	21. For patients undergoing bone marrow transplantation, the Mini nutritional assessment (MNA) and the subjective global assessment (SGA) are recommended for nutritional evaluation.^30^	Level 5	A
	22. Recommended nutritional intake prior to HSCT: Energy: 35–50 kcal/kg/day; protein: 1.5–2.0 g/kg/day; No specific recommendation for glucose; adjustment of fatty acid composition: ① Saturated fatty acids: < 7%–10% of total daily calorie intake (based on cardiovascular risk assessment); ② Monounsaturated fatty acids: 15% of total daily calorie intake; ③ polyunsaturated fatty acids: 5%–10% of total daily calorie intake.^2^ (The upper limit of energy recommendation should be reserved for patients with severe malnutrition or hypermetabolic states to prevent clinical misapplication.)	Level 5	B
	23. The fluid requirement for HSCT patients is based on the recommendation for healthy individuals, which is 35 ml/kg/day. However, adjustments may be necessary in these calculations due to dynamic losses and frequent fluid retention.^2^	Level 5	B
	24. Nutritional counseling must be tailored to the patient’s nutritional requirements while also considering dietary restrictions, cultural and religious characteristics, as well as dietary patterns such as vegetarian, omnivorous, vegan, kosher, and others.^2^	Level 5	A
Psychological support	25. Better psychological adaptation outcomes are associated with patients who demonstrate relatively intact cognitive assessment, higher perceived personal control, and approach-oriented coping styles. For individuals exhibiting maladaptive responses, interventions such as cognitive restructuring, psychoeducation, and emotion acceptance can effectively modify related psychological mechanisms, thereby enhancing overall adjustment.^31^	Level 1	A
	26. Avoidant coping styles, cognitive appraisals of HSCT as a threat, and loss of self-efficacy are predictors of psychological distress.^31^	Level 1	A
	27. Relaxation-only or avoidance-focused strategies may be less effective than interventions involving active emotional processing, and relaxation training should be used as an adjunct rather than the sole core component.^31^	Level 1	A
	28. The efficacy of interventions based on cognitive behavioral therapy (CBT) or incorporating active emotional processing components is well supported.^31^	Level 1	A
	29. Mindfulness-based interventions are effective in alleviating fatigue. Complementary and alternative therapies such as yoga, acupuncture, acupressure, auricular therapy, and massage therapy may also be utilized to improve fatigue in patients.^18^	Level 1	B
	30. Interventions that involve substantial psychological input and active therapist engagement are generally more effective compared to those with minimal psychological focus or those that are primarily self-directed.^31^	Level 1	B
	31. Delivering psychological interventions in a group format can help reduce the burden on resources and lower economic costs.^31^	Level 1	A
Precautionary considerations	32. When face-to-face care delivery is impeded, telehealth may serve as a feasible and effective alternative.^17^	Level 1	B
	33. Assessment and reassessment can be conducted during hospitalization, in a day-care unit, or in an outpatient setting, depending on the patient’s condition and transplantation schedule.^29^	Level 5	A
	34. A flexible approach to prehabilitation supervision is recommended. For patients who initially follow a guided but unsupervised training program but show poor adherence, transitioning to a supervised format is advisable. Conversely, the reverse adjustment may also be considered.^29^	Level 5	A

Evidence levels were assigned according to the JBI evidence hierarchy. Recommendation strength was determined by the expert panel based on the FAME principles, considering evidence level, clinical applicability, feasibility, appropriateness, meaningfulness, and effectiveness. Therefore, recommendation grade does not solely reflect evidence level.

## Discussion

### Multidisciplinary team building

The multidimensional complexity of trimodal prehabilitation dictates that a single discipline is insufficient to adequately address the patient’s physiological, nutritional, and psychological needs. Consequently, establishing a multidisciplinary team is pivotal for enhancing intervention efficacy. Existing evidence indicates that multidisciplinary prehabilitation for HSCT patients has demonstrated clear benefits in improving walking capacity, weight management, and self-reported functional status.^17^ The physician is responsible for overall condition control and judgment of intervention contraindications, the physical therapist formulates the exercise program, the dietitian addresses nutritional needs, the psychologist focuses on the emotional state, and the specialized nurse is responsible for the whole-process implementation and feedback. The linkage of various roles can avoid the limitations of single intervention.^17^^,^^33^ Furthermore, the implementation of regular team meetings facilitates a dynamic response to changes in the patient’s condition and intervention response, and is more conducive to achieving information exchange and timely identification of intervention contradictions. For example, regarding the nutritional consumption of patients after exercise, dietitians and physical therapists need to collaboratively adjust the plan, thereby preventing the rigid application of standardized protocols. Assessment and program design led by clinicians with specialized training not only align with clinical guideline standards but also mitigate medical risks associated with prehabilitation, ensuring intervention safety.^17^ However, the construction of multidisciplinary teams in medical centers in less developed regions still needs to be strengthened. Poor communication between disciplines and unclear division of responsibilities may become practical obstacles to the achievement of goals.^16^ Furthermore, current literature identifies the pre-transplantation period as the optimal window to initiate these interventions, with earlier initiation correlating with more significant post-operative functional recovery and potentially shorter hospital stays.^17^^,^^29^ A proposed underlying mechanism is that early intervention effectively preserves skeletal muscle mass and enhances cardiopulmonary functional reserve, thereby establishing a physiological foundation for patients to better tolerate transplantation-related therapies and achieve accelerated post-operative recovery.^6^^,^^7^

### Exercise prescription

Exercise intervention serves as a core component of the trimodal prehabilitation for HSCT patients. Within this framework, individualized assessment is the prerequisite for delivering precise interventions. Studies indicate significant individual variability among HSCT patients, where factors such as age, pre-existing physical activity levels, disease treatment regimen, and nutritional status may all influence prehabilitation outcomes.^6^ Conducting a pre-transplant assessment to accurately identify high-risk individuals and their core intervention needs can optimize the utilization of limited healthcare resources and minimize the risks associated with under- or over-intervention.^35^ The development of an exercise prescription should be performed by an exercise specialist with oncology-specific training. This process should be based on a comprehensive evaluation of the patient’s physical function, clinical and pathological characteristics, and lifestyle, integrated with evidence-based exercise guidelines and written instructions to formulate a personalized plan. This approach aims to minimize exercise-related risks and enhance patient adherence.^17^ One possible explanation is that physical therapists can accurately assess patients’ exercise capacity and movement disorders, thus preventing unreasonable exercise programs such as excessive training and improper exercise patterns from non-professional evaluations. Standardized written instructions can improve the accuracy of implementation by patients and caregivers, avoid the ambiguity of verbal guidance, and facilitate the adjustment of programs based on written records during follow-up, thereby enhancing the continuity of intervention. However, in view of the shortage of physical therapists in some clinical settings,^36^^,^^37^ the feasibility of trained hematology specialized nurses assisting in completing basic assessments and delivering written instructions deserves consideration in the future. Furthermore, given the generally compromised baseline physical condition of HSCT patients, the implementation of an exercise program must adhere to the principle of progressive overload. Exercise duration and intensity should be gradually increased according to the patient’s tolerance to avoid exercise-related injuries, exacerbated fatigue, and even abandonment of rehabilitation due to discomfort, which may reduce intervention adherence or lead to intervention discontinuation.^5^^,^^29^^,^^33^

In this study, the exercise regimen for HSCT patients incorporated various modalities such as warm-up, aerobic exercise, resistance training, stretching, and cool–down activities. Patients were encouraged to select specific exercise forms based on their individual fitness levels, personal preferences, and the availability of home exercise space and equipment. This emphasis on variety and enjoyment is recognized as a key factor in supporting long-term adherence.^17^^,^^18^^,^^35^ For example, home-based patients can formulate implementable home exercise programs (such as resistance band training and indoor walking), and patients with a sports background can appropriately increase the intensity, which is consistent with their living scenarios and improves adherence.

During the implementation of exercise interventions, intensity can be monitored through quantified rating scales or target heart rate zones, utilizing a combination of subjective perception and objective metrics. This includes assessing exercise intensity based on patient heart rate or employing tools such as the Modified Borg Scale for self-reported exertion to improve the accuracy of assessment.^5^^,^^17^^,^^18^^,^^29^^,^^33^ For example, home-based patients can conduct self-assessment using the Modified Borg Scale and adjust the intensity in combination with heart rate monitoring during outpatient follow-up; for patients with unstable conditions, heart rate monitoring should be given priority to control the intensity, so as to avoid deviations in subjective judgment and reflect the principle of safety first. A critical consideration is that a RCT by Wood et al.^34^ demonstrated low feasibility for home-based high-intensity aerobic interval training. This finding suggests that healthcare professionals should tailor exercise program design to individual patient characteristics and streamline monitoring procedures to enhance long-term adherence. In addition, HSCT patients are physically weak and have limited energy. Complex monitoring procedures (such as frequent recording and complex instrument operation) will reduce patients’ willingness to participate, and convenient monitoring methods may be beneficial to improving adherence.^34^ Future prehabilitation practice may benefit from adopting flexible supervision strategies, where patients who do not adhere to an initially prescribed unsupervised, guided program could be transitioned to a supervised format, and vice versa.^29^ In view of the situation that patients are not proficient in operating intelligent devices, paper record forms can be designed to be more simple and easy to understand, convenient to fill in, so as to improve the accessibility of monitoring.

### Nutritional optimization

Studies indicate that nutritional status serves as a critical foundation for HSCT patients to tolerate the transplantation process and facilitate postoperative recovery. Even minor changes in body weight may hold clinical significance.^17^ As patients are prone to issues such as anorexia, gastrointestinal dysfunction, and increased protein catabolism during transplantation, proactive nutritional optimization prior to the procedure plays a positive role in mitigating adverse effects and enhancing treatment tolerance.^2^^,^^38^ This study adopted the specific nutritional consensus developed by Barban et al.^2^ for patients undergoing HSCT, and set the pre-transplant energy intake recommendation at 35–50 kcal/kg/day to enhance preoperative nutritional reserve. In contrast, the European Society for Clinical Nutrition and Metabolism (ESPEN) 2021 guideline on clinical nutrition recommends an energy intake of 25–30 kcal/kg/day for general cancer patients.^39^ The ESPEN guideline states that if individual measurement of total energy expenditure is unavailable, the patient’s energy requirement can be assumed to be similar to that of healthy individuals. The discrepancy in recommended thresholds may stem from differences in target populations and intervention purposes. Patients scheduled for HSCT frequently face challenges such as inflammatory stress, hypermetabolism, and hypercatabolism, resulting in a special demand for nutritional reserve.^2^ Accordingly, the upper limit of this energy recommendation is more suitable for patients with severe malnutrition or hypermetabolic status. In view of the above differences, individualized adjustment should be made in clinical practice according to patients’ nutritional risk, physical condition and specific transplantation protocol. Evidence shows that providing written information and personalized nutritional therapy via regular phone or video consultations by healthcare professionals including dietitians, along with thorough clarification and discussion of dietary care with patients and caregivers, helps address practical concerns promptly and prevents intervention deviations due to information asymmetry.^2^ Incorporating caregivers into the nutritional management system helps standardize home dietary management and avoid dietary misunderstandings such as over-nourishment caused by traditional experience.^40^ Dietitians understand patients’ dietary preferences through interviews, communicate dietary preparation skills with caregivers, and jointly formulate implementable home dietary plans to ensure the continuity of nutritional management and avoid intervention deviations caused by dietary inconsistency with patients’ habits.^2^ Furthermore, nutritional assessment should be initiated within 30 days before the patient is admitted to the hospital, so that there is sufficient time to improve the patient’s nutritional status through nutritional intervention, avoid slow post-operative recovery and increased complications caused by insufficient pre-transplant nutrition.^2^^,^^30^ For patients at high nutritional risk (such as those with weight loss and poor appetite), their nutritional status changes rapidly, so the assessment interval should be shortened (≤ 15 days) to timely detect nutritional deterioration and adjust the intervention plan; for patients without nutritional risk, their nutritional status is relatively stable, so the assessment interval can be extended to avoid excessive assessment increasing the medical burden.^2^ Additionally, healthcare providers should ensure that nutritional counseling considers individual dietary restrictions, cultural or religious practices, and food preferences. At the same time, it is necessary to give priority to ensuring nutritional intake, avoid nutritional deficiency caused by excessive accommodation of preferences, and balance adherence with nutritional needs.^2^ For example, there are significant dietary differences in different regions of China: people in the south prefer rice, while those in the north prefer pasta. Nutritional guidance should be adapted to regional dietary characteristics. For vegetarian populations, key guidance should be provided on how to supplement protein through plant-based foods to avoid nutritional deficiency. For patients with religious dietary taboos, alternative ingredients should be selected to meet nutritional needs without violating the taboos.

### Psychological support

Patients with hematologic malignancies facing HSCT are often at risk for cognitive dysfunction due to multiple factors such as advanced age, prior chemotherapy, physical deconditioning, and fatigue.^13^ The interplay between physical discomfort and psychological distress can further exacerbate the treatment burden.^41^, ^42^, ^43^ From a risk prediction perspective, pre-transplant avoidant coping strategies, inadequate professional emotional and informational support, perceived threats related to the disease and future, and a common state of diminished agency among HSCT candidates are all associated with higher levels of psychological distress and somatic symptoms.^44^^,^^45^

Evidence confirms that interventions based on CBT or incorporating active emotional processing components, delivered through methods such as cognitive restructuring, psychoeducation, and coping skills training, can help patients break avoidant coping patterns and enhance their ability to manage anxiety, depression, and psychosocial distress.^31^^,^^46^^,^^47^ This finding may be related to cognitive restructuring helping patients understand the transplantation process and treatment side effects, relieve fear from the unknown and form positive views on treatment outcomes, as well as emotional acceptance allowing patients to face anxiety, depression and other negative emotions squarely and avoid aggravated psychological problems caused by suppression. Furthermore, Intensive, professionally led interventions demonstrate significantly superior outcomes compared to low-intensity or self-directed approaches, supporting the conclusion that complex psychological distress is difficult to address through self-regulation alone.^31^^,^^48^ In response to the shortage of clinical psychologists in less developed regions, clinical practice may consider proposing a collaborative intervention model of “psychologist + specialized nurse”. Psychologists are responsible for formulating personalized intervention plans, while specialized nurses are responsible for daily follow-up and guiding patients to implement the plans, so as to improve the accessibility and effectiveness of intervention.^40^ Importantly, a professionally guided framework does not exclude patient autonomy; the two can complement each other across different clinical contexts. For example, mindfulness-based interventions, which do not require continuous professional involvement and can be practiced independently by patients, focus on engaging patients in active symptom management. These approaches not only effectively alleviate fatigue but also improve self-efficacy, making them well-suited to patients’ daily needs.^18^^,^^49^ However, care should be taken to avoid positioning relaxation training as a core intervention, because it focuses on “avoiding” negative emotions (such as relieving anxiety through relaxation) rather than facing and solving the root causes of anxiety. If used as a core intervention for a long time, it may invisibly strengthen negative coping tendencies and reduce the overall intervention effect.^31^^,^^50^ It can be considered as an auxiliary intervention method to relieve acute anxiety and fatigue, such as short-term relaxation training to alleviate emotions when patients feel nervous before transplantation. It is worth noting that the group intervention model can alleviate patients’ loneliness through peer support, help them learn coping experiences through communication, improve their psychological adaptability, reduce the overall intervention cost, ease the resource burden, and is suitable for large-scale promotion.^31^ However, unlike general oncology group interventions, group intervention models for HSCT patients should comply with infection control requirements. Moreover, for HSCT patients, such psychological interventions need to be adapted to the transplant treatment process: for example, interventions can be delivered via telehealth during the isolation period to avoid infection risks, and the intensity and frequency of interventions should be adjusted according to the patient’s hematological parameters and physical tolerance.

### Implications for nursing practice

From the perspective of the integrated evidence, several key challenges exist in translating trimodal prehabilitation into clinical practice for HSCT patients. In response to the above problems, it is recommended to optimize resource allocation through multi-party collaboration (e.g., training hematology nurses as primary implementers of prehabilitation), strengthening public and patient education regarding pre-transplant rehabilitation, developing standardized nursing protocols, and establishing a seamless rehabilitation system covering both the pre- and post-transplant periods. Combined with the increasingly mature telemedicine technology, remote development of HSCT prehabilitation can be promoted to improve the accessibility of intervention, which is particularly suitable for primary medical centers and home-based patients. Notably, as core coordinators of the multidisciplinary team, nurses serve as a communication bridge between different professionals and ensure smooth engagement with patients.^16^ This role is critical for integrating exercise, nutritional, and psychological interventions into a coherent and unified trimodal prehabilitation program. Importantly, clinical nursing should prioritize individualization based on evidence-based principles, adjusting interventions according to each patient’s clinical condition, preferences, and circumstances. For instance, for allogeneic transplant recipients, interventions must accommodate long-term immunosuppression and protective isolation requirements. Telehealth may be prioritized for exercise guidance and psychological support to minimize infection risk. In contrast, autologous transplant candidates benefit from more flexible approaches, including home-based exercise programs with progressive intensity and family-engaged nutritional support, ensuring prehabilitation interventions are both safe and effective.

### Limitations

This study has several limitations in synthesizing the best evidence for trimodal prehabilitation in patients undergoing HSCT. First, there is room for improvement regarding the authority and comprehensiveness of the evidence sources, as guidelines with high recommendation grades constituted a relatively small proportion of the included literature. It is worth noting that several items with low-level evidence were assigned Grade A recommendation strength. These ratings were determined with consideration of expert consensus and clinical applicability in addition to evidence level. Second, evidence specifically addressing psychological prehabilitation before transplantation requires further enrichment and refinement. Third, since most of the included studies originated from diverse countries and regions, variations exist in healthcare resource allocation and clinical protocols compared to the actual context of primary care institutions in China. Therefore, to facilitate the translation of this evidence into clinical practice, future efforts should optimize its applicability by integrating factors such as China’s cultural background, specific patient characteristics, and local healthcare resource conditions, thereby supporting the enhancement of treatment tolerance and long-term outcomes in HSCT patients.

## Conclusions

This study conducted a systematic search of domestic and international literature on trimodal prehabilitation management in HSCT. The evidence was synthesized across five domains: multidisciplinary team building, exercise prescription, nutritional optimization, psychological support, and precautionary considerations, resulting in the formulation of 34 evidence statements. This work aims to provide an evidence-based foundation to support clinical practice for nursing professionals. It is recommended that when implementing these findings, nurses carefully consider the local healthcare context and select the most appropriate evidence for translation into practice, with the aim of enhancing the quality of care.

## CRediT authorship contribution statement

Danni Wu: Conceptualization, Methodology, Writing - Original Draft. Limei Xue: Data Curation, Formal Analysis, Visualization. Lingzhi Xu: Data Curation, Formal Analysis, Visualization. Jie Pan: Data Curation, Formal analysis. Tao Chen: Data Curation, Formal analysis. Yan Wang： Data Curation, Formal analysis. Sufang Zhao: Supervision, Writing – Review & Editing. All authors have read and approved the final manuscript.

## Ethics statement

Not required.

## Data availability statement

The data that support the findings of this study are available from the corresponding author, upon reasonable request.

## Declaration of generative AI and AI-assisted technologies in the writing process

No AI tools/services were used during the preparation of this work.

## Funding

This study received no external funding.

## Declaration of competing interest

The authors declare no conflict of interest.
