# Supplementary material for: Trimodal prehabilitation for hematopoietic stem cell transplantation: A best evidence summary
Source: Asia Pac J Oncol Nurs. 2026 Jun 12;13:100993. doi: 10.1016/j.apjon.2026.100993 (PMC13320315; doi:10.1016/j.apjon.2026.100993)
Supplement: Multimedia component 1 [file mmc1.docx]

| **Database** | **Search strategies** | **Results** |
| --- | --- | --- |
| PubMed | #1："Hematopoietic Stem Cell Transplantation"[MeSH Terms] OR "Bone Marrow Transplantation"[MeSH Terms] OR "Peripheral Blood Stem Cell Transplantation"[MeSH Terms] OR "Umbilical Cord Blood Stem Cell Transplantation"[MeSH Terms] OR "hematopoietic stem cell transplant*"[Title/Abstract] OR "HSCT"[Title/Abstract] OR "bone marrow transplant*"[Title/Abstract] OR "allogeneic transplant*"[Title/Abstract] OR "autologous transplant*"[Title/Abstract]  #2："Prehabilitation"[MeSH Terms] OR "Preoperative Care"[MeSH Terms] OR "Exercise"[MeSH Terms] OR "Diet"[MeSH Terms] OR "Nutrition Therapy"[MeSH Terms] OR "Mindfulness"[MeSH Terms] OR "Meditation"[MeSH Terms] OR "Relaxation Therapy"[MeSH Terms] OR "Cognitive Behavioral Therapy"[MeSH Terms] OR "Patient Education"[MeSH Terms] OR "prehabilitation"[Title/Abstract] OR "pre-rehabilitation"[Title/Abstract] OR "exercise therapy"[Title/Abstract] OR "physical activit*"[Title/Abstract] OR "walk*"[Title/Abstract] OR "yoga"[Title/Abstract] OR "nutrition support"[Title/Abstract] OR "dietary supplement*"[Title/Abstract] OR "mindfulness"[Title/Abstract] OR "meditation"[Title/Abstract] OR "music therap*"[Title/Abstract] OR "aromatherap*"[Title/Abstract] OR "CBT"[Title/Abstract] OR "psychological intervention"[Title/Abstract]  #3："Clinical Decision Making"[MeSH Terms] OR "Practice Guidelines"[MeSH Terms] OR "Meta Analysis"[Publication Type] OR "Systematic Review"[Publication Type] OR "Randomized Controlled Trial"[Publication Type] OR "consensus"[Title/Abstract] OR "guideline"[Title/Abstract] OR "recommend*"[Title/Abstract] OR "evidence-based practice"[Title/Abstract] OR "meta analysis"[Title/Abstract] OR "systematic review"[Title/Abstract] OR "evidence summar*"[Title/Abstract] OR "RCT"[Title/Abstract]  #4：#1 AND #2 AND #3 | 410 |
| Web of Science | #1: "(TS=(""Hematopoietic Stem Cell Transplantation"" OR ""Bone Marrow Transplantation"" OR ""Peripheral Blood Stem Cell Transplantation"" OR ""Umbilical Cord Blood Stem Cell Transplantation"") OR TI=(""hematopoietic stem cell transplant*"" OR ""HSCT"" OR ""bone marrow transplant*"" OR ""allogeneic transplant*"" OR ""autologous transplant*"") OR AB=(""hematopoietic stem cell transplant*"" OR ""HSCT"" OR ""bone marrow transplant*"" OR ""allogeneic transplant*"" OR ""autologous transplant*"")) and Preprint Citation Index (Exclude-Database) and Research Commons (Exclude-Database)"  #2: "(TS=(""Prehabilitation"" OR ""Preoperative Care"" OR ""Exercise"" OR ""Diet"" OR ""Nutrition Therapy"" OR ""Mindfulness"" OR ""Meditation"" OR ""Relaxation Therapy"" OR ""Cognitive Behavioral Therapy"" OR ""Patient Education"") OR TI=(""prehabilitation"" OR ""pre-rehabilitation"" OR ""exercise therapy"" OR ""physical activit*"" OR ""walk*"" OR ""yoga"" OR ""nutrition support"" OR ""dietary supplement*"" OR ""mindfulness"" OR ""meditation"" OR ""music therap*"" OR ""aromatherap*"" OR ""CBT"" OR ""psychological intervention"") OR AB=(""prehabilitation"" OR ""pre-rehabilitation"" OR ""exercise therapy"" OR ""physical activit*"" OR ""walk*"" OR ""yoga"" OR ""nutrition support"" OR ""dietary supplement*"" OR ""mindfulness"" OR ""meditation"" OR ""music therap*"" OR ""aromatherap*"" OR ""CBT"" OR ""psychological intervention"")) and Preprint Citation Index (Exclude-Database) and Research Commons (Exclude-Database)"  #3: "(TS=(""meta-analysis"" OR ""systematic review"" OR ""randomized controlled trial"" OR ""guideline"") OR TS=(""clinical decision making"" OR ""practice guidelines"" OR ""consensus"" OR ""guideline"" OR ""recommend*"" OR ""evidence-based practice"" OR ""meta-analysis"" OR ""systematic review"" OR ""evidence summar*"" OR ""RCT"") OR TI=(""consensus"" OR ""guideline"" OR ""recommend*"" OR ""evidence-based practice"" OR ""meta-analysis"" OR ""systematic review"" OR ""evidence summar*"" OR ""RCT"") OR AB=(""consensus"" OR ""guideline"" OR ""recommend*"" OR ""evidence-based practice"" OR ""meta-analysis"" OR ""systematic review"" OR ""evidence summar*"" OR ""RCT"")) and Preprint Citation Index (Exclude-Database) and Research Commons (Exclude-Database)"  #4: "#3 AND #2 AND #1 and Preprint Citation Index (Exclude-Database) and Research Commons (Exclude-Database)" | 555 |
| Cochrane Library | ("Hematopoietic Stem Cell Transplantation" OR "Bone Marrow Transplantation" OR "Peripheral Blood Stem Cell Transplantation" OR "Umbilical Cord Blood Stem Cell Transplantation" OR "hematopoietic stem cell transplant*" OR "HSCT" OR "bone marrow transplant*" OR "allogeneic transplant*" OR "autologous transplant*") AND ("Prehabilitation" OR "Preoperative Care" OR "Exercise" OR "Diet" OR "Nutrition Therapy" OR "Mindfulness" OR "Meditation" OR "Relaxation Therapy" OR "Cognitive Behavioral Therapy" OR "Patient Education" OR "prehabilitation" OR "pre-rehabilitation" OR "exercise therapy" OR "physical activit*" OR "walk*" OR "yoga" OR "nutrition support" OR "dietary supplement*" OR "mindfulness" OR "meditation" OR "music therap*" OR "aromatherap*" OR "CBT" OR "psychological intervention") AND ("Clinical Decision Making" OR "Practice Guidelines" OR "consensus" OR "guideline" OR "recommend*" OR "evidence-based practice" OR "meta-analysis" OR "systematic review" OR "evidence summar*" OR "RCT" OR "Randomized Controlled Trial") | 7 |
| CINAHL | MH ((MH "Hematopoietic Stem Cell Transplantation+") OR (MH "Bone Marrow Transplantation+") OR (MH "Peripheral Blood Stem Cell Transplantation") OR (MH "Umbilical Cord Blood Stem Cell Transplantation") OR (TI hematopoietic stem cell transplant* OR AB hematopoietic stem cell transplant* OR TI HSCT OR AB HSCT OR TI "bone marrow transplant*" OR AB "bone marrow transplant*" OR TI "allogeneic transplant*" OR AB "allogeneic transplant*" OR TI "autologous transplant*" OR AB "autologous transplant*")) AND MH ((MH "Prehabilitation+") OR (MH "Preoperative Care+") OR (MH "Exercise+") OR (MH "Diet+") OR (MH "Nutrition Therapy+") OR (MH "Mindfulness+") OR (MH "Meditation+") OR (MH "Relaxation Therapy+") OR (MH "Cognitive Behavioral Therapy+") OR (MH "Patient Education+") OR (TI prehabilitation OR AB prehabilitation OR TI pre-rehabilitation OR AB pre-rehabilitation OR TI "exercise therapy" OR AB "exercise therapy" OR TI "physical activit*" OR AB "physical activit*" OR TI walk* OR AB walk* OR TI yoga OR AB yoga OR TI "nutrition support" OR AB "nutrition support" OR TI "dietary supplement*" OR AB "dietary supplement*" OR TI mindfulness OR AB mindfulness OR TI meditation OR AB meditation OR TI "music therap*" OR AB "music therap*" OR TI "aromatherap*" OR AB "aromatherap*" OR TI CBT OR AB CBT OR TI "psychological intervention" OR AB "psychological intervention")) AND MH ((MH "Clinical Decision Making+") OR (MH "Practice Guidelines+") OR (PT "Meta Analysis") OR (PT "Systematic Review") OR (PT "Randomized Controlled Trial") OR (TI consensus OR AB consensus OR TI guideline OR AB guideline OR TI recommend* OR AB recommend* OR TI "evidence-based practice" OR AB "evidence-based practice" OR TI "meta-analysis" OR AB "meta-analysis" OR TI "systematic review" OR AB "systematic review" OR TI "evidence summar*" OR AB "evidence summar*" OR TI RCT OR AB RCT)) | 170 |
| CNKI | TKA=('造血干细胞移植' + '骨髓移植' + '外周血干细胞移植' + '脐血移植') AND TKA=('预康复' + '运动' + '活动' + '训练' + '散步' + '慢跑' + '瑜伽' + '按摩' + '气功' + '饮食' + '营养' + '膳食补充剂' + '心理' + '正念' + '冥想' + '感恩日记' + '芳香疗法' + '音乐疗法' + '认知行为疗法' + '教育') AND TKA=('临床决策' + '指南' + '标准' + '建议' + '共识' + '意见' + '推荐' + 'Meta分析' + '荟萃分析' + '系统评价' + '证据总结' + '循证护理实践' + '随机对照研究') | 290 |
| Wanfang Data | (主题 =("造血干细胞移植" OR "骨髓移植" OR "外周血干细胞移植" OR "脐血移植"))AND(主题 =("预康复" OR "运动" OR "活动" OR "训练" OR "散步" OR "慢跑" OR "瑜伽" OR "按摩" OR "气功" OR "饮食" OR "营养" OR "膳食补充剂" OR "心理" OR "正念" OR "冥想" OR "感恩日记" OR "芳香疗法" OR "音乐疗法" OR "认知行为疗法" OR "教育"))AND(主题 =("临床决策" OR "指南" OR "标准" OR "建议" OR "共识" OR "意见" OR "推荐" OR "Meta 分析" OR "荟萃分析" OR "系统评价" OR "证据总结" OR "循证护理实践" OR "随机对照研究")) | 158 |
| SinoMed | M=(造血干细胞移植 OR 骨髓移植 OR 外周血干细胞移植 OR 脐血移植) AND M=(预康复 OR 运动 OR 活动 OR 训练 OR 散步 OR 慢跑 OR 瑜伽 OR 按摩 OR 气功 OR 饮食 OR 营养 OR 膳食补充剂 OR 心理 OR 正念 OR 冥想 OR 感恩日记 OR 芳香疗法 OR 音乐疗法 OR 认知行为疗法 OR 教育) AND M=(临床决策 OR 指南 OR 标准 OR 建议 OR 共识 OR 意见 OR 推荐 OR Meta 分析 OR 荟萃分析 OR 系统评价 OR 证据总结 OR 循证护理实践 OR 随机对照研究) | 21 |
